# Artificial intelligence-assisted phase recognition and skill assessment in laparoscopic surgery: a systematic review

**DOI:** 10.3389/fsurg.2025.1551838

**Published:** 2025-04-11

**Authors:** Wenqiang Liao, Ying Zhu, Hanwei Zhang, Dan Wang, Lijun Zhang, Tianxiang Chen, Ru Zhou, Zi Ye

**Affiliations:** ^1^ Department of General Surgery, RuiJin Hospital LuWan Branch, Shanghai Jiaotong University School of Medicine, Shanghai, China; ^2^ Hangzhou Institute for Advanced Study, University of Chinese Academy of Sciences, Hangzhou, China; ^3^ Institute of Intelligent Software, Guangzhou, China; ^4^ Institute of Software Chinese Academy of Sciences, Beijing, China; ^5^ School of Cyber Space and Technology, University of Science and Technology of China, Hefei, China

**Keywords:** laparoscopic surgery, phase recognition, skill evaluation, methods, surgical datasets

## Abstract

With the widespread adoption of minimally invasive surgery, laparoscopic surgery has been an essential component of modern surgical procedures. As key technologies, laparoscopic phase recognition and skill evaluation aim to identify different stages of the surgical process and assess surgeons’ operational skills using automated methods. This, in turn, can improve the quality of surgery and the skill of surgeons. This review summarizes the progress of research in laparoscopic surgery, phase recognition, and skill evaluation. At first, the importance of laparoscopic surgery is introduced, clarifying the relationship between phase recognition, skill evaluation, and other surgical tasks. The publicly available surgical datasets for laparoscopic phase recognition tasks are then detailed. The review highlights the research methods that have exhibited superior performance in these public datasets and identifies common characteristics of these high-performing methods. Based on the insights obtained, the commonly used phase recognition research and surgical skill evaluation methods and models in this field are summarized. In addition, this study briefly outlines the standards and methods for evaluating laparoscopic surgical skills. Finally, an analysis of the difficulties researchers face and potential future development directions is presented. Moreover, this paper aims to provide valuable references for researchers, promoting further advancements in this domain.

## 1 Introduction

**Figure 1:**
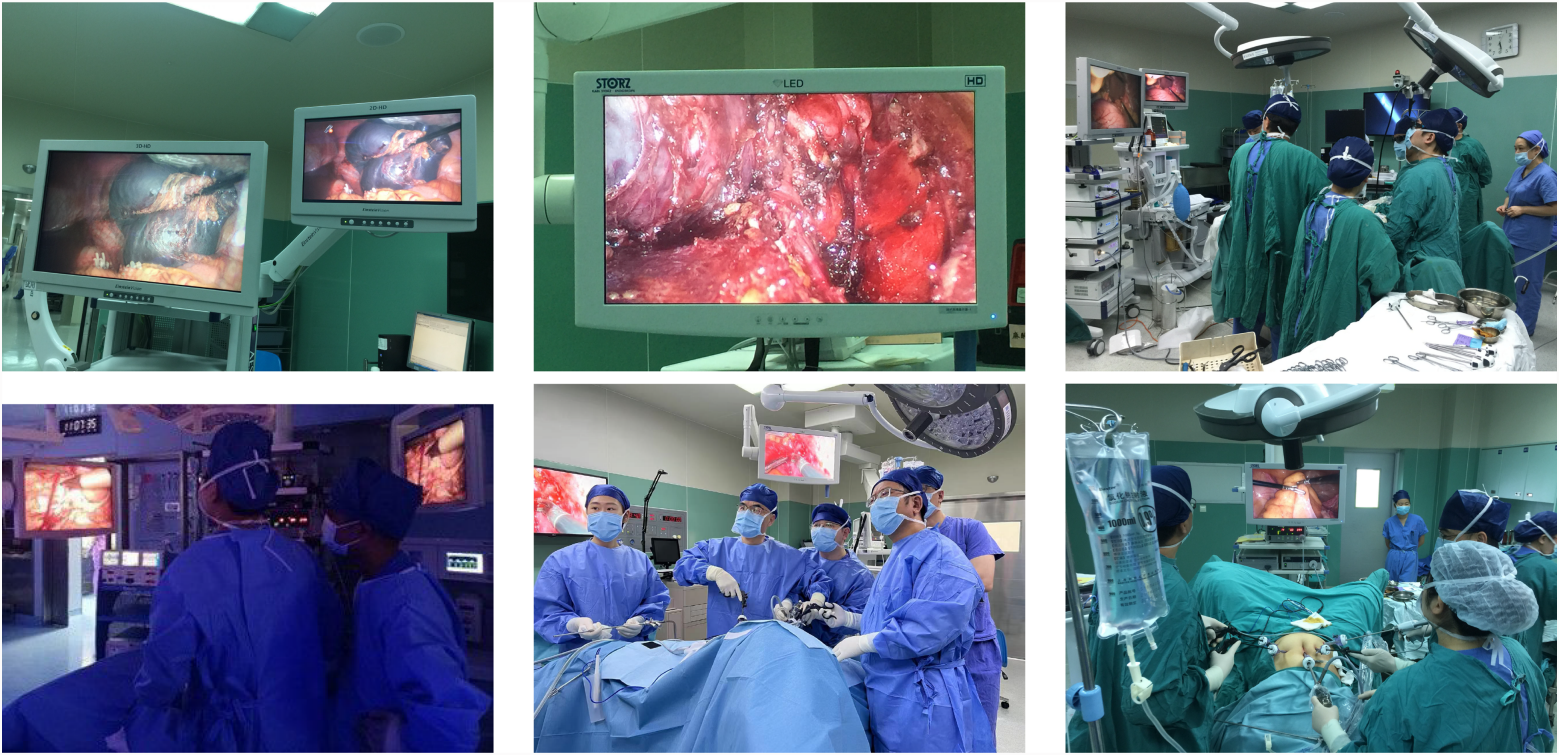
Clinical workflow of laparoscopic surgery: Real-time endoscopic view monitored by the surgical team.

As an advanced minimally invasive surgical technique, laparoscopic surgery has been widely applied in a variety of procedures. This approach involves making multiple small incisions in different areas of the patient’s abdomen, through which a camera and various specialized surgical instruments are inserted (1). Surgeons manipulate these instruments while monitoring the surgical field through a high-definition display, As shown in Figure 1. Therefore, it significantly reduces the trauma surgery causes, shortens the patient’s postoperative recovery time, and lowers postsurgical pain. However, laparoscopic surgery requires high surgical skills from surgeons, especially in understanding the surgical process (2), interpreting information from the surgical field (3), adapting to complex surgical scenarios (4), and mastering precise operational skills (5).

Laparoscopic surgery has several advantages over traditional open surgery, including reduced pain, reduced patient recovery time, decreased wound infections, and reduced morbidity and mortality (6). In addition, laparoscopic surgery provides an enlarged high-definition surgical field of view (7), which can significantly improve surgical accuracy. However, this also places greater demands on surgeons, who must have extensive surgical experience and proficiency with surgical tools. At the same time, laparoscopic surgery has higher requirements for surgical equipment and instruments, often requiring high-resolution camera equipment and specialized surgical tools, which are costly and require regular maintenance. Although laparoscopic surgery has many advantages, it also has certain limitations. Patient selection is one of the main challenges. For example, laparoscopic surgery is often very challenging for patients who have previously undergone open abdominal surgery (6), while traditional open surgery has fewer limitations in these cases and even advantages when addressing complex procedures.

**Figure 2:**
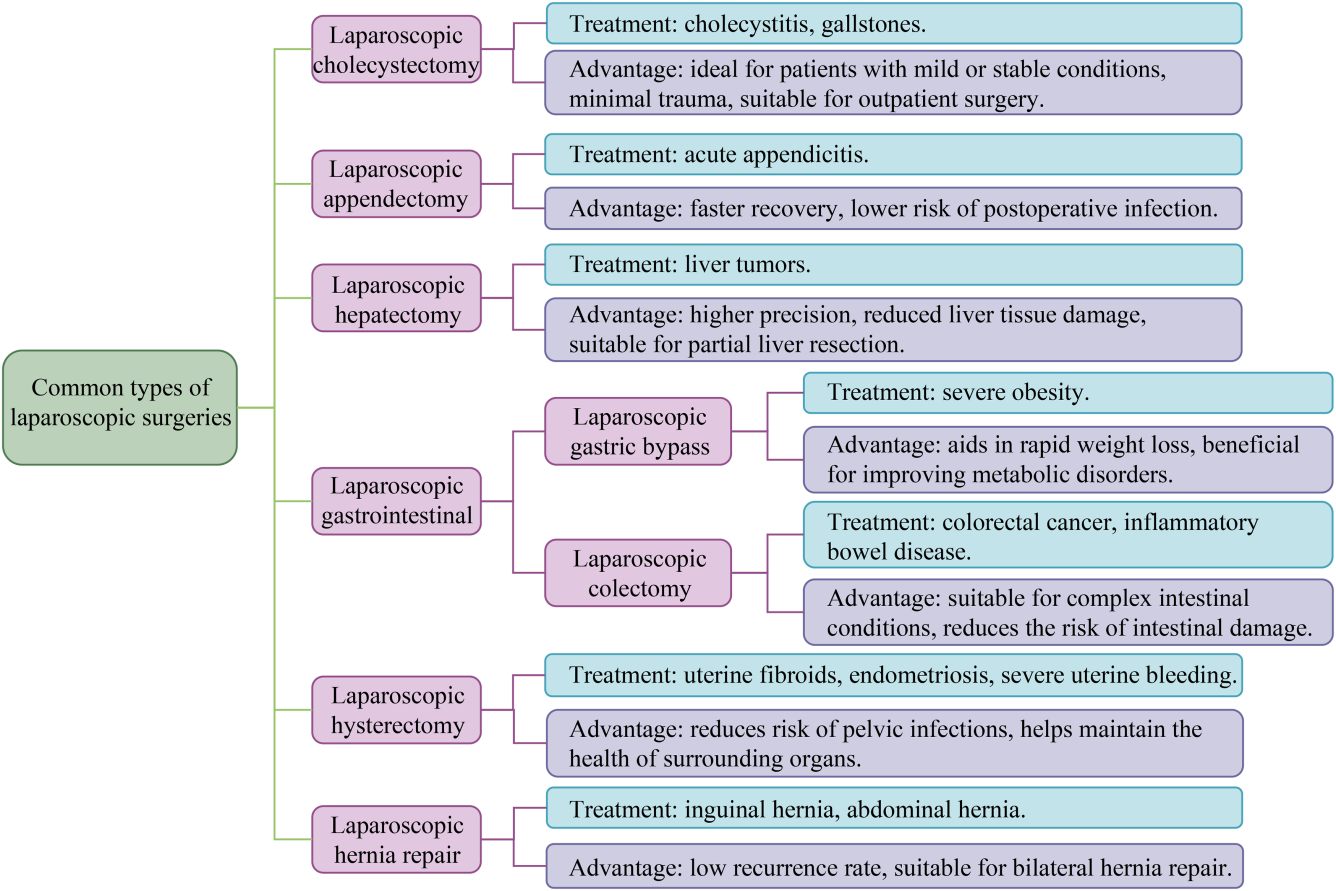
Common types of laparoscopic surgeries. All laparoscopic surgeries have the advantages of minimal trauma, fast recovery, short hospital stay, mild postoperative pain, and low risk of complications. The figure shows the main indications and unique advantages of various laparoscopic surgeries.

Common types of laparoscopic surgery include laparoscopic cholecystectomy, appendectomy, hepatectomy, gastrointestinal surgery, and hysterectomy. Each type of surgery has its specific indications. At the same time, most of the advantages of laparoscopy for these procedures are reflected in the ability to reduce postoperative discomfort, accelerate patient recovery time, and reduce the risk of infection by incision (7). The details of the common types of laparoscopic surgery are presented in Figure 2, which presents the specific indications corresponding to each type of surgery and the advantages of laparoscopic performing these procedures.

Phase recognition is the analysis of surgical videos to identify different stages of surgery, which is vital to understanding the surgical process, providing intraoperative assistance, evaluating the performance of surgeons (8), and predicting the remaining surgical time. Accurate phase recognition can give surgeons real-time feedback and issue warnings in abnormal situations, improving the safety and effectiveness of surgery. Skill assessment in laparoscopic surgery comprehensively evaluates the surgeon’s operational skills during surgery (9). Skill assessment contributes to determining whether the surgeon’s operations satisfy practical standards, providing targeted feedback and improvement suggestions for the surgeon. The intrinsic relationship between stage identification and skill assessment is mainly reflected in three aspects as follows: Firstly, accurate stage identification provides a necessary contextual framework for calculating meaningful surgical skill assessment indicators, as the performance of surgeons can only be correctly evaluated at specific surgical stages. Secondly, emerging hybrid architectures demonstrate that utilizing shared spatiotemporal features and jointly training two tasks can improve accuracy compared to isolated models. In addition, real-time phase recognition triggers a skill assessment protocol for specific stages, achieving situational awareness assessment that considers different technical requirements during the surgical stage. Accurate stage identification provides reliable foundational data for skill assessment, making the assessment process more precise. Meanwhile, skill assessment results can be better adapted to the complexity of actual surgery by continuously optimizing recognition algorithms, thus promoting improved phase recognition.

In current research on surgical video analysis, beyond the task of phase recognition, there are various other tasks, including the detection of the use of surgical tools, the segmentation of surgical instruments, the segmentation of organs, the recognition of surgical maneuvers, and the prediction of the remaining surgical duration (10). While surgical video analysis encompasses multiple technical tasks such as instrument segmentation and organ detection, phase recognition and skill assessment hold particular clinical primacy for two key reasons: Firstly, real-time phase recognition directly realizes the intraoperative decision support system, while skill assessment provides actionable feedback for surgical training, both of which meet the key requirements of surgical quality control. Secondly, instrument detection and tissue segmentation are often the foundation of advanced phase analysis, rather than the ultimate goal itself. Related studies have indicated that understanding the temporal evolution of surgical tool usage patterns and uncovering their relationships with respective surgical phases can provide vital clues for recognizing laparoscopic surgery phase recognition (11). However, sometimes, due to the presence of blood in the surgical tools or differences in the color of the tool shafts, depending on the surgical tools for phase recognition can result in decreased accuracy (3). Although there is a close association between surgical tool detection and surgical phase recognition, the latter focuses mainly on the overall progress and stages of surgery. In contrast, the detection of surgical tools involves the identification and localization of specific tools. Both tasks contribute to enhancing the safety and efficiency of surgical operations. Recognizing surgical phases and maneuvers is vital in strengthening surgical skills, efficiency, and safety by providing feedback to surgeons (8). However, surgical phase recognition aims to categorize each video frame into high-level stages of the surgery (12). By contrast, action recognition aims to dissect each video frame into fine-grained and meticulous tasks directly from the data. Surgical phase recognition requires more extended video frames than action recognition since each phase typically encompasses several actions (8). Compared with other visual recognition and classification tasks, laparoscopic surgery phase recognition poses a more daunting challenge due to the high visual similarity of frames across different phases. Moreover, modeling the inter-phase correlations presents significant challenges (13). The main challenges in this field include the following aspects: firstly, blood contamination of the lens may occur during the surgical process, causing occlusion (14); commonly, electrocautery can produce smoke; and motion blur caused by rapid camera movement or instrument adjustment can reduce frame clarity. Secondly, subtle differences between consecutive surgical stages can lead to fuzzy classification. In addition, skill assessment tasks may be subjective due to the reliance on expert annotations for skill assessment, which may vary among raters. Addressing these challenges requires robust algorithms that can handle noisy inputs and capture contextual temporal patterns.

This review aims to systematically revisit the latest research advances in phase recognition and skill assessment in laparoscopic surgery, considering the importance of laparoscopic surgery in modern healthcare and the potential of phase recognition and skill assessment to improve the quality and safety of surgical procedures. We aim to explore public datasets, existing model methodologies, practical applications, challenges faced, and potential future directions in laparoscopic surgery phase recognition and skill assessment. In addition, this provides references for researchers and surgeons in the field, fostering further development and application of laparoscopic surgery technology.

The main contributions of our study are listed below:

• This review investigates laparoscopic surgery’s phase recognition and skill assessment research, providing a comprehensive analysis and organization of these methods.
• We compile a summary of research on laparoscopic surgery phase recognition using public datasets and perform performance comparisons of these methods. In addition, we provide an in-depth analysis of the limitations of existing datasets, including annotation inconsistencies, lack of multi-center validation, and challenges in cross-domain generalization.
• We identified common features of high-performing methods, including commonly used spatial models, temporal models, and other optimization strategies. Furthermore, we compare the evolution of temporal modeling techniques (LSTM, TCN, Transformer) and discuss their advantages and limitations, providing insights into the future direction of phase recognition model architectures. On this basis, we also delved into optimization strategies such as attention mechanisms, transfer learning, federated learning, and multimodal fusion. These are the biggest differences between our review and other existing reviews.
• After analyzing the research methods and application areas associated with laparoscopic surgery phase recognition and skill assessment, we summarized the main challenges and potential opportunities for future development in this field.

## 2 Literature search and selection methods

### 2.1 Search strategy

A systematic literature search was conducted across three primary platforms: Google Scholar, arXiv, and Sci-Hub, focusing on studies published between January 2019 and January 2025 to capture the latest advancements in deep learning (DL) applications for laparoscopic surgery. Conducted searches by combining the keywords “laparoscopic surgery” or “minimally invasive surgery” with “phase recognition,” “surgical phase analysis,” or “surgical skill assessment” to ensure comprehensive coverage of the relevant research domain. By applying methodological filtering criteria, the search incorporates technical aspects such as “deep learning,” “computer vision,” or “neural networks,” along with studies involving “datasets,” “Cholec80,” “M2CAI16,” or “benchmarks” to ensure the retrieval of research that includes representative data and evaluation standards.

### 2.2 Inclusion and exclusion criteria

During the research screening process, a series of standards are strictly followed to ensure the relevance and scientific validity of the selected literature. The research needs to focus specifically on laparoscopic surgeries such as cholecystectomy or appendectomy, and adopt deep learning based methods for surgical stage identification or skill assessment. Meanwhile, data transparency is an important consideration in screening, requiring research to use publicly available datasets or provide detailed descriptions of proprietary datasets. In addition, the research needs to be published in peer-reviewed journals or conferences, or as a preprint for arXiv, and ensure that the full text is provided in English.

To ensure the rigor of screening, certain types of research are excluded. For example, research involving non laparoscopic surgery is not considered, and studies using traditional image processing, rule-based systems, or non machine learning methods also do not meet screening criteria. Meanwhile, research without quantitative results or complete method descriptions will not be included. In addition, studies using datasets from unknown sources were also excluded to ensure the reliability of the selected literature.

## 3 Publicly available surgical datasets

### 3.1 Cholec80

The Cholec80 (14) endoscopic video dataset contains 80 videos of cholecystectomy surgeries performed by 13 surgeons. In addition, captured at a rate of 25 frames per second (fps) and downsampled to 1 fps for processing, these videos have resolutions of 1,920×1,080 or 854×480. The dataset provides annotations for two tasks: surgical phase recognition (7 phases) and binary tool presence detection (10). Table 1 displays the specific phases of the dataset. Each video is annotated by a single senior surgeon, with phases P2 (Calot triangle dissection) and P4 (Gallbladder dissection) containing the highest frame counts, while P5 (Gallbladder packaging) and P7 (Gallbladder retraction) are the shortest.

**Table 1:** Summary of public datasets on phase recognition in laparoscopic surgery.

Name	Year	Data	Procedure	Number of phases	Phases
Cholec80	2016	80 videos	Cholecystectomy	7	P1: Preparation
					P2: Calot Triangle Dissection
					P3: Clipping and Cutting
					P4: Gallbladder Dissection
					P5: Gallbladder Packaging
					P6: Cleaning and Coagulation
					P7: Gallbladder Retraction
M2CAI16	2016	41 videos	Cholecystectomy	8	P1: Trocar Placement
					P2: Preparation
					P3: Calot Triangle Dissection
					P4: Clipping and Cutting
					P5: Gallbladder Dissection
					P6: Gallbladder Packaging
					P7: Cleaning and Coagulation
					P8: Gallbladder Retraction
AutoLaparo	2018	21 videos	Hysterectomy	7	P1: Preparation
					P2: Dividing Ligament and Peritoneum
					P3: Dividing Uterine Vessels and Ligament
					P4: Transecting the Vagina
					P5: Specimen Removal
					P6: Suturing
					P7: Washing

While Cholec80 is considered large-scale in terms of case quantity (80 videos), it exhibits limitations in three key dimensions to the extent that we consider it to be medium-scale: Firstly, in terms of annotation granularity, the tools on the Cholec80 dataset only label based on its presence (≥50% visibility of tooltips), lacking pixel level segmentation or motion data. Secondly, compared to other public datasets, the Cholec80 dataset has a clear disadvantage in that all surgical videos come from a single institution with standardized procedures and lack diversity. Finally, subsampling at 1 fps results in significantly fewer total frames compared to other public datasets, limiting the ability for temporal modeling. Additionally, it is important to note that a single annotation mechanism designed with only one surgeon for annotation may introduce subjective bias in the definition of phase transitions, particularly in the fuzzy intervals between similar phase transitions (such as P5–P6 transitions). This may affect the model generalization ability between datasets with different annotation protocols.

### 3.2 M2CAI16-workflow

In this study, the surgical workflow challenge dataset, M2CAI16-workflow, was created for the M2CAI challenge. Forty-one laparoscopic videos of cholecystectomy, each with a resolution of 1,920×1,080 and recorded at a speed of 25 frames per second (15), were included in the dataset. Skilled surgeons separated each video into eight phases (16), with Table 1 providing comprehensive descriptions of each step. The surgical phases defined in the M2CAI16-workflow dataset are similar to those specified in the Cholec80 dataset, i.e., one more “Trocar Placement” phase is determined before the seven phases described in the Cholec80 dataset.

Compared with other data sets for laparoscopic surgery phase recognition, the labels in the MACAI16 workflow data set are phase labels defined in collaboration with multiple authoritative institutions, high in quality, and suitable for model training and validation of surgical phase recognition. But there are also certain limitations. Firstly, compared to the 80 surgical videos in the Cholec80 dataset, MACAI16-workflow only contains 41 videos, which is relatively insufficient in data volume and challenging to support large-scale deep learning models’ training fully. In addition, the data of the MACAI16 flow only involve cholecystectomy surgery and do not cover the stages of other laparoscopic surgical procedures, which limits the generalizability of the model. More importantly, compared to other datasets for laparoscopic surgery phase recognition, MACAI16-workflow only annotates surgical stages, lacking tools, anatomical structures, or other multi-task information, which appears incomplete in multi-task learning scenarios.

### 3.3 AutoLaparo

The dataset AutoLaparo (2) is a large-scale, integrated, multi-task data set for image-guided surgical automation in laparoscopic hysterectomy. This dataset was developed based on complete videos of the entire hysterectomy procedure. The 21 videos in the dataset were recorded at a speed of 25 frames per second with a resolution of 1,920×1,080 pixels. The dataset defines three highly correlated tasks: surgical workflow recognition, laparoscopic motion prediction, and instrument and key anatomical segmentation. Experienced senior gynecologists and experts performed annotations. In Table 1, the specific stage definitions of the dataset are visible. In summary, it can be concluded that P2 and P3 occupy a more significant proportion of the videos, while P1 and P5 account for a smaller proportion.

The AutoLaparo dataset is one of the few designed explicitly for laparoscopic hysterectomy surgery. Still, it only includes hysterectomy surgery, limiting its generalizability to other types of laparoscopic surgery scenarios. In addition, there are only 21 videos in the AutoLaparo dataset. Although each video has a longer duration, the number is limited, making it difficult to cover multiple surgical variants and complex situations. However, the reason why it is called a large-scale dataset is from a comprehensive and multi-faceted perspective. Although it only contains 21 videos, each video contains more total frames than the Cholec80 dataset and has three common annotation tasks, including surgical phase recognition, surgical tool segmentation, and laparoscopic motion prediction. In addition, the annotations were jointly validated by senior gynecologists from 7 hospitals to ensure cross institutional consistency.

### 3.4 JIGSAWS

JIGSAWS (17) (JHU-ISI Gesture and Skill Assessment Working Set) is a dataset used for studying surgical skill assessment and surgical activity modeling, created in collaboration between Johns Hopkins University (JHU) and Intuitive Surgical Inc. (ISI). The JIGSAWS dataset mainly includes three basic surgical tasks, namely suturing, knot-tying, and needle-passing. Eight surgeons with different levels of experience completed these three tasks, repeating each task five times to provide rich skill performance data. The JIGSAWS dataset consists of kinematic data, video data, and manual annotation, covering multiple dimensions of surgical operations and providing important resources for automated assessment of surgical skills.

In terms of skill assessment, JIGSAWS adopts a standardized scoring system, which is evaluated by an experienced gynecologist based on the OSATS (Objective Structured Assessment of Technical Skills) scoring system. The scoring system covers six core dimensions: respect for tissue, suture/needle handling, time and motion, flow of operation, overall performance, and final product quality. Each criterion is rated on a scale from 1 to 5, and the evaluation process adopts blind testing to reduce subjective bias. The dataset emphasizes the comparison of skill levels between novices and experts, ranging from resident physicians with less than 10 h of experience to experts with over 100 h of experience, providing researchers with continuous skill level data that helps explore the development of surgical skills and optimize automated evaluation methods.

However, despite the significant value of the JIGSAWS dataset in surgical skill assessment, there are still certain limitations. This dataset mainly focuses on basic training tasks such as suturing and knotting, without involving more complex clinical procedures, which limits its applicability in real surgical environments. Therefore, although JIGSAWS provides a standardized experimental framework in the field of surgical skill assessment, its coverage still needs to be further expanded in the future to support more complex surgical scenarios and more comprehensive skill assessment research.

## 4 Research on phase recognition in laparoscopic surgery videos

### 4.1 Definition and importance of phase recognition

Laparoscopic video phase recognition uses video analysis techniques to automatically identify and classify different surgical process stages. This involves analyzing laparoscopic video to detect phases during surgery automatically. Understanding every step of the surgical workflow is the goal of assisting different applications, such as auxiliary surgery and postoperative analysis.

Phase recognition is essential for surgical workflow analysis because it is helpful for the standardization and postoperative evaluation of procedures (18). Phase recognition is also crucial to improve the safety (19) and surgery efficiency. It can monitor the surgical procedure, alert physicians to possible problems before they arise (4), help physicians better prepare for the next operation or make decisions, guarantee the procedure’s success, and support surgical education and analysis. Furthermore, a more objective assessment of the surgeon’s skill can be made by analyzing the key steps in the surgical video.

### 4.2 Exploration of phase recognition methods and technologies

Much literature has been accumulated on the study of phase recognition in laparoscopic surgery, which has similarities and significant differences. To better understand the development of this field and the current situation, we will start with two aspects of commonalities and differences in these studies, focusing on in-depth analysis through direct objectives, core steps, and adopted model. Through comparative analysis of these aspects, we hope to help researchers in this field have a clearer understanding of current research trends and challenges, promoting further development of phase recognition research in laparoscopic surgery.

#### 4.2.1 Analysis based on research objectives

**Figure 3:**
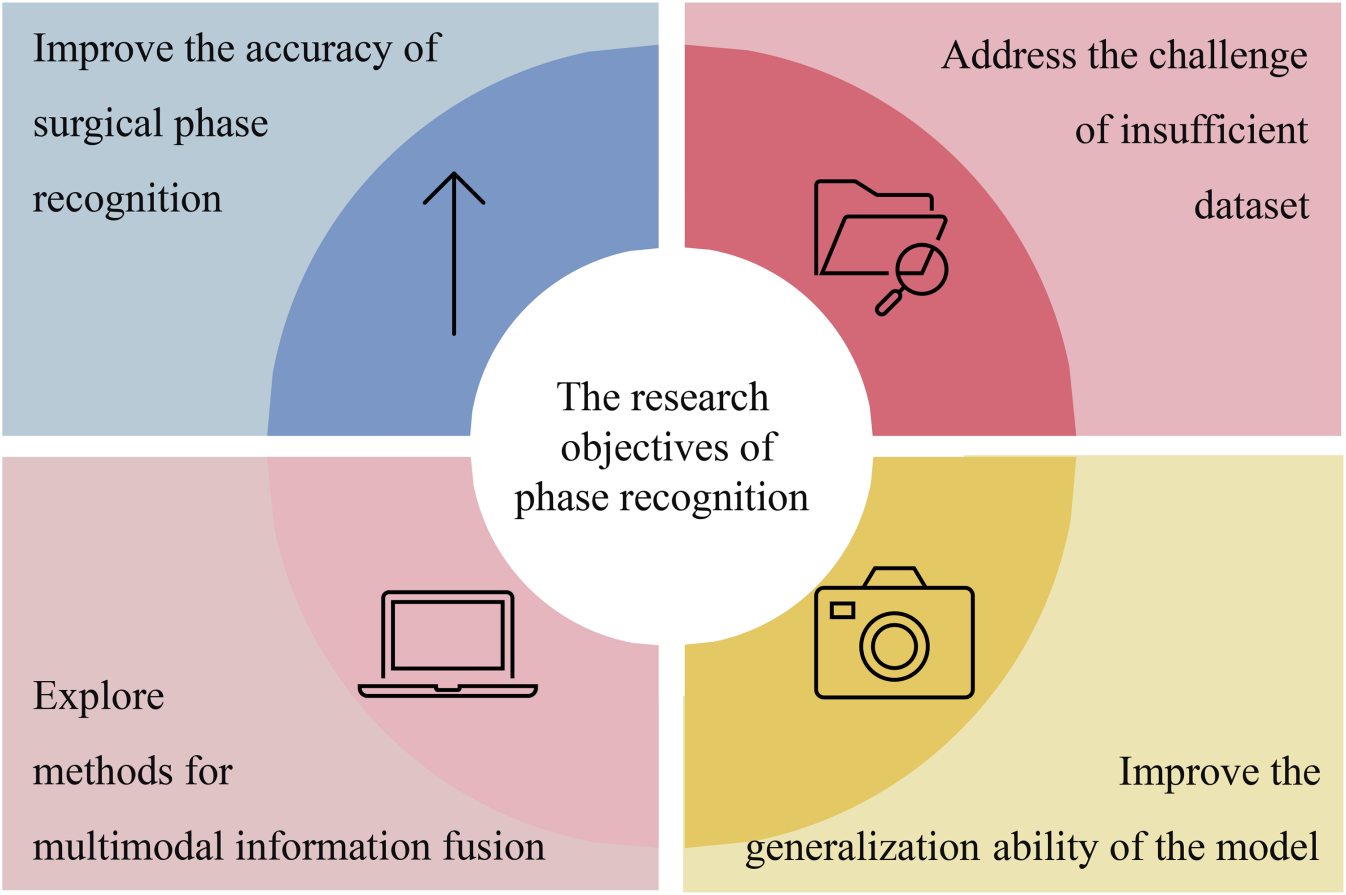
Summarizing the research objectives related to laparoscopic surgery phase recognition.

In the research on laparoscopic surgery phase recognition, the direct objectives of researchers vary. As illustrated in Figure 3, these objectives focus on four key areas: improving the accuracy and efficiency of phase recognition of laparoscopic surgery, addressing the challenges of insufficient datasets, exploring methods for multimodal information fusion, and improving the generalizability of the methods used in phase recognition tasks.

Most studies focus on improving existing models to enhance the accuracy and efficiency of laparoscopic surgery phase recognition. For example, Ding et al. (4) achieved improved precision in laparoscopic surgery phase recognition by extracting high-level features from surgical videos. This method improves the model’s performance by correcting for blurriness or incorrect predictions resulting from low-level frames.

The lack of enough datasets is another difficulty for researchers in phase recognition. Many researchers have proposed corresponding solutions. For example, in (20), because of the insufficient annotated data, the authors used semi-supervised learning to improve the model’s performance. Furthermore, in (21), the federated learning method was proposed, which allowed the model to train on multiple dispersed datasets. This protects data privacy and will enable data to be used by several institutions.

Multimodal information fusion is also essential in the phase recognition task of laparoscopic surgery. Combining tool recognition features with phase recognition features is one of the applications of the method. For example, by adding tool recognition results as auxiliary features to the phase recognition model, Yuan et al. (22) improved the phase prediction accuracy of the model.

Another goal of phase recognition is to improve the model’s generalization ability on different surgical environments or datasets. Therefore, they used data augmentation and transfer learning techniques to ensure the model can maintain stable performance. In (20), the model’s generalization ability was improved by increasing the diversity of training data, that is, by using data augmentation techniques. In (23), the model’s generalization ability and accuracy have been significantly improved by pre-training on one surgical type and transferring knowledge to other surgical types.

#### 4.2.2 Analysis based on core steps

Although the direct goals of researchers vary, the core steps of laparoscopic surgery phase recognition research are generally similar. Figure 5 shows that the main steps of laparoscopic surgery phase recognition research include the collection and preprocessing of laparoscopic surgery videos, phase classification annotation, deep learning model training, and testing, as well as model performance evaluation.

In the study of phase recognition in laparoscopic surgical videos, the first step is to collect a large number of laparoscopic surgical videos. These videos can come from the same medical center or many different medical centers. These videos are preprocessed, including format conversion and de-identification, to protect patients’ privacy. The video content is annotated following different stages of surgery, providing basic data for training deep learning models.

To accurately train AI models, researchers collaborated with experienced surgeons to define the key stages of surgery and provided corresponding detailed annotations for the videos. The number and content of the key stages represented in these studies are different, and the corresponding numbers of key stages for each study are summarized in Tables 2 and 3. Although the number of these stages varies, their definitions are consistent in key steps such as Calot triangulation, tissue separation, and cutting. Some studies even annotate possible adverse events that may occur during the surgical process with the purpose of training models to recognize these events. For example, the laparoscopic cholecystectomy video dataset constructed by Tomer Golany et al. (43) specifically recorded adverse events such as significant bleeding, gallbladder perforation, and massive bile leakage, providing valuable annotated data for model training. Using annotated data, researchers can train deep-learning models to recognize and predict surgical phases in laparoscopic videos.

**Table 2:** Comparison of studies on surgical phase recognition tasks using DL models-Part 1.

Ref.	Year	Type	Dataset	Number of Phases	DL model
(24)	2019	Single-task	M2CAI16	8	ResNet50 + LSTM
			Cholec80	7	
(25)	2019	Multi-task	Cholec80	7	CNN + LSTM
(26)	2019	Single-task	NPA	7	CNN + LSTM
(27)	2019	Single-task	Cholec80	7	CNN
			CATARACTS	4	
(28)	2019	Multi-task	Cholec80	7	CNN + NARX
(29)	2019	Multi-task	NPA	11	Inception-ResNet-v2 + LightGBM
(1)	2020	Single-task	NPA	8	ResNet50
(30)	2020	Single-task	NPA	7	CNN + Non-local Block
(31)	2020	Single-task	Cholec80	7	ResNet50 + MS-TCN
			NPA		
(32)	2020	Single-task	NPA	9	CNN
(33)	2020	Multi-task	NPA	7	InceptionV3 + ResNet50
				8	
(34)	2020	Multi-task	Cholec80	7	CNN + LSTM
(5)	2021	Single-task	Cholec80	7	CNN + GNN
(10)	2021	Single-task	Cholec80	7	CNN + LSTM + SSM
			NPA	13	
(35)	2021	Single-task	M2CAI16	8	ResNeXt101 + SE
(16)	2021	Single-task	Cholec80	7	Transformer
			M2CAI16	8	
(36)	2021	Multi-task	Cholec80	7	IIM + MS-TCN
(37)	2021	Single-task	Cholec80	7	PeleeNet + ST-ERFNet
(18)	2021	Single-task	NPA	6	CNN + LSTM
(23)	2021	Single-task	NPA	7	Conv1D + LSTM
(38)	2021	Single-task	NPA	11	ResNet50 + TCN
(39)	2021	Single-task	Cholec80	7	CNN + LSTM + 3D-CNN
			NPA	21	
(40)	2021	Single-task	NPA	8	3DCNN
(41)	2021	Single-task	NPA	7	SVM + HMM
(42)	2021	Single-task	NPA	8	IPCSN + MS-TCN + PKNF
(19)	2022	Single-task	Cholec80	7	CNN + CBAM + IndyLSTM
(2)	2022	Multi-task	AutoLaparo	7	SV-RCNet
					TMRNet
					TeCNO
					Trans-SVNet
(3)	2022	Single-task	NPA	7	EfficientNet-B7 + SAM
(43)	2022	Single-task	NPA	10	ResNet50 + MS-TCN

NPA = not publicly available.

**Table 3:** Comparison of studies on surgical phase recognition tasks using DL models-Part 2.

Ref.	Year	Type	Dataset	Number of Phases	DL model
(4)	2022	Single-task	Cholec80	7	ResNet50 + RCDL + SFE + SFA
			M2CAI16	8	
(7)	2022	Multi-task	Actions 160	16	CNN + LSTM + TCN
			Cataract-101	10	
			Cholec80	7	
(44)	2022	Single-task	Cholec80	7	ResNet + TCN + GRU + Causal TCN
			M2CAI16	8	
(22)	2022	Multi-task	Cholec80	7	ResNet50 + UNet + TeCNO + MS-TCN
(45)	2022	Single-task	NPA	7	CNN + LSTM + HMM
(46)	2022	Single-task	NPA	12	Resnet50 + MSTCN
					Resnet50 + Trans-SVNet
(47)	2022	Single-task	NPA	5	Conv3D + seq2seq
(48)	2022	Single-task	Cholec80	7	CNN + LSTM
			NPA	12	
(49)	2022	Single-task	NPA	8	TCN + LSTM
(8)	2023	Single-task	Cholec80	7	L-Trans + G-Informer
			AutoLaparo	7	
(50)	2023	Single-task	NPA	12	FCN + MS-TCN
(51)	2023	Single-task	NPA	6	VTN + LSTM
(52)	2023	Multi-task	Cholec80	7	Transformer
			M2CAI16	8	
(53)	2023	Single-task	Cholec80	7	EfficientNetV2 + Transformer
(54)	2023	Single-task	Cholec80	7	Attn_conv Inc_2DLSTM + Gcaps_TAE
(55)	2023	Single-task	Cholec80	7	Swin Transformer + LSTM
(56)	2023	Single-task	Cholec80	7	ResNet50 + MS-TCN + ASFormer
(21)	2023	Single-task	NPA	6	ResNet50
(57)	2023	Single-task	Cholec80	7	CNN + TCN + GRU
			NPA	10	
(58)	2023	Single-task	NPA	6	YOLOv3
					EfficientNet-B7
(20)	2023	Multi-task	Cholec80	7	MoCo v2
					SimCLR
					DINO
					SwAV
(59)	2023	Single-task	NPA	7	CNN + Transformer
(60)	2023	Single-task	NPA	13	DESM
(61)	2023	Single-task	NPA	5	ResNet50 + SS-TCN
			CATARACTS	11	
(62)	2023	Multi-task	NPA	7	ASFormer + TCN
(63)	2024	Single-task	Cholec80	7	ResNet + MS-TCN
(64)	2024	Single-task	Cholec80	7	Faster R-CNN + ResNet + Transformer
			M2CAI16	8	
			Autolaparo	7	
(65)	2024	Single-task	Cholec80	7	Transformer + Hierarchical Temporal Attention
			Autolaparo	7	
(66)	2025	Single-task	Cholec80	7	Vision Transformer + L-Trans + G-Informer
			AutoLaparo	7	

NPA = not publicly available.

In the deep learning model training and testing process, laparoscopic surgery phase recognition research mainly includes four core steps: video processing and feature extraction, temporal modeling, design of classification layers, and construction of loss functions. In addition, in specific applications, multimodal fusion techniques may also be involved further to enhance the performance and robustness of the model. To facilitate understanding of the subsequent model analysis, let us first outline these key steps and the underlying principles.

Firstly, in laparoscopic surgery phase recognition, it is necessary to convert the input video frame sequence into feature representations that deep learning models can effectively process. Assuming that the surgical video contains T frames, each frame can be represented as $X_{t}$, where t∈{1,2,…,T}. These frames are input into a deep neural network (such as a Convolutional Neural Network, CNN) for feature extraction. Specifically, as shown in Equation (1), the feature extraction process can be represented as:

(1) $$F_{t}=\text{CNN}(X_{t})$$

Among them, $F_{t}$ is the feature vector extracted from the t-th frame. The entire video can be transformed into a series of feature vectors, denoted $\left\{F_{1},F_{2},\ldots ,F_{T}\right\}$. The CNN here can be replaced with other spatial models.

Next, due to the surgical stage’s apparent temporal continuity and interdependence, the feature sequences $\left\{F_{1},F_{2},\ldots ,F_{T}\right\}$ will be input into the temporal model for modeling. Temporal models can capture dynamic features and long-term and short-term dependencies during surgical procedures. Taking the LSTM temporal model as an example, when using LSTM for time modeling, it can be represented by the following formula (2):

(2) $$h_{t}=\text{LSTM}(F_{t},h_{t-1})$$

Among them, $h_{t}$ is the hidden state of the LSTM model in frame t, which depends on the current input feature $F_{t}$ and the previous hidden state $h_{t-1}$. The LSTM here can be replaced with other temporal models.

The output hidden state $h_{t}$ of the temporal model will be input into a fully connected layer or classifier to predict the surgical phase label for each frame. The calculation process of probability distribution is given by Equation (3):

(3) $$P(y_{t}|X)=\text{softmax}(Wh_{t}+b)$$

Where W and b are the classifier’s weight matrix and bias vector, and $y_{t}$ is the predicted phase label of the t-th frame. Calculating the phase label probability at each time step and maximizing it, the phase sequence of the entire video can be obtained.

In order to optimize model performance, the cross-entropy loss function is commonly used during the training process to measure the error between predicted labels and true labels. The loss function is specifically defined by Equation (4):

(4) $$L=-\frac{1}{T}\sum_{t=1}^{T}\sum_{c=1}^{C}y_{t}^{\left(c\right)}\log\;P(y_{t}^{\left(c\right)}|X)$$

where C is the total number of phase categories, $y_{t}^{\left(c\right)}$ is the one-hot encoding of the true label, and $P(y_{t}^{\left(c\right)}|X)$ is the predicted probability for category c at time step t. The model can better match the predicted probabilities to the true labels across all frames by minimizing the cross-entropy loss.

When phase categories are highly imbalanced, the standard cross-entropy loss may be dominated by high-frequency classes. Focal Loss dynamically adjusts sample weights to focus on hard-to-classify examples, as shown by Equation (5):

(5) $$L_{FL}=-\frac{1}{T}\sum_{t=1}^{T}\sum_{c=1}^{C}\alpha_{t}^{\left(c\right)}{\left(1-P(y_{t}^{\left(c\right)}|X)\right)}^{\gamma }y_{t}^{\left(c\right)}\log\;P(y_{t}^{\left(c\right)}|X)$$

where $\alpha_{t}^{\left(c\right)}$ is a weighting factor for class c at time step t, used to balance class frequency. γ is the focusing parameter that adjusts the contribution of easy and hard samples.

In order to ensure the continuity of predictions between adjacent frames and avoid unreasonable phase jumps, Temporal Consistency Loss is often used in research. The formula is given by Equation (6):

(6) $$L_{TC}=\frac{1}{T-1}\sum_{t=1}^{T-1}{\left\|P(y_{t}|X)-P(y_{t+1}|X)\right\|}^{2}$$

This loss minimizes the change in prediction probability between adjacent frames, making the phase recognition results smoother, thereby improving the temporal stability and logical coherence of the surgical process.

In addition to the above process, in some complex scenarios, multimodal feature fusion technology can be introduced to improve the accuracy of phase recognition. For example, other features, such as tool usage, can also be integrated into visual features. The fusion method can be achieved through feature concatenation or weighted summation. Feature concatenation can be represented by Equation (7):

(7) $$F_{t}^{\prime }=\text{concat}(F_{t},G_{t})$$

where $F_{t}$ represents the visual features and $G_{t}$ represents the tool features. The weighted summation method can be expressed by Equation (8):

(8) $$F_{t}^{\prime }=\alpha F_{t}+\beta G_{t}$$

Among them, α and β are learnable weight parameters that balance the contributions of different modal features.

These steps and methods constitute the basic process and principles of phase recognition research in laparoscopic surgery, providing a theoretical basis for further analysis and optimization of specific models.

#### 4.2.3 Analysis based on models or methods

Based on the above analysis, we will currently discuss the most complex aspect of this field: the commonalities and differences among the models or methods applied in laparoscopic surgery phase recognition research. Table 4 summarizes the studies conducted on public datasets for laparoscopic surgery phase recognition. Most of these studies are based on laparoscopic cholecystectomy and primarily utilize the Cholec80 and M2CAI16-workflow datasets. As shown in this table, we can observe that the performance of these methods is related to the used models and the improvements made to the techniques.

**Table 4:** Comparison of related research on phase recognition using public datasets.

Ref.	Year	Application^a^	DL model	Dataset	Accuracy
(24)	2019	Phase recognition	ResNet50 + LSTM	M2CAI16	91.2%
				Cholec80	92.4%
(25)	2019	Phase recognition	CNN + LSTM	Cholec80	89.2%
		Tool recognition			
(30)	2020	Phase recognition	CNN + Non-local Block	Cholec80	91.7%
(31)	2020	Phase recognition	ResNet50 + MS-TCN	Cholec80	88.56%
(5)	2021	Phase recognition	CNN + GNN	Cholec80	93.77%
(10)	2021	Phase recognition	CNN + LSTM + SSM	Cholec80	90.8%
(35)	2021	Phase recognition	ResNeXt101 + SE Attention	M2CAI16	85.8%
(15)	2021	Workflow recognition	TMRNet	Cholec80	90.1%
				M2CAI16	87%
(16)	2021	Phase recognition	Trans-SVNet	Cholec80	90.3%
				M2CAI16	87.2%
(67)	2021	Phase recognition	CNN + SE Attention	Cholec80	91.26%
(36)	2021	Workflow recognition	IIM + MS-TCN	Cholec80	88%
		Instrument detection			
(37)	2021	Phase recognition	PeleeNet + ST-ERFNet	Cholec80	86.07%
(19)	2022	Phase recognition	ResNet50 + CBAM + IndyLSTM	Cholec80	89.8%
(4)	2022	Phase recognition	ResNet50 + RCDL + SFE + SFA	Cholec80	91.8%
				M2CAI16	91.6%
(7)	2022	Phase recognition	CNN + LSTM + TCN	Cholec80	90.2%
		Video retrieval task			
(68)	2022	Phase recognition	CDC Networks	M2CAI16	91.4%
(13)	2022	Phase recognition	CNN + Transformer	Cholec80	89.27%
(44)	2022	Phase recognition	TCN + GRU	Cholec80	92%
				M2CAI16	88.2%
(8)	2023	Phase recognition	L-Trans + G-Informer	Cholec80	91.5%
				AutoLaparo	81.43%
(52)	2023	Phase recognition	Transformer + VFE + FE + LSC	Cholec80	93.12%
		Tool recognition		M2CAI16	91.5%
(69)	2023	Phase recognition	Self-KD	Cholec80	93.24%
(53)	2023	Phase recognition	EfficientNetV2 + Transformer	Cholec80	94.9%
(54)	2023	Phase recognition	CNN + LSTM + BiGRU	Cholec80	98.95%
(55)	2023	Workflow recognition	Swin Transformer + LSTM	Cholec80	92.8%
(56)	2023	Phase recognition	ResNet50 + MS-TCN + ASFormer	Cholec80	95.43%
(63)	2024	Phase recognition	ResNet + MS-TCN	Cholec80	93.6%
(64)	2024	Phase recognition	Faster R-CNN + ResNet + Transformer	Cholec80	93.5%
				M2CAI16	91.8%
				Autolaparo	81.6%
(65)	2024	Phase recognition	Transformer + Hierarchical Temporal Attention	Cholec80	93.4%
				Autolaparo	85.7%
(66)	2025	Phase recognition	Vision Transformer + L-Trans + G-Informer	Cholec80	92.4%
				AutoLaparo	81.4%

Most of the studied surgical procedures are laparoscopic cholecystectomies.

^a^Phase recognition: Focuses on segmenting surgical procedures into distinct stages (e.g., gallbladder dissection). Workflow recognition: Encompasses broader process analysis.

The research and development of phase recognition in laparoscopic surgery has gradually evolved from spatial to temporal models. The earliest research mainly focused on using the spatial features of static images for classification. Researchers mostly use traditional computer vision methods or direct use of convolutional neural networks (CNN) for surgical image classification. For example, in 2019, Gurvan Lecuyer et al. (27) proposed a CNN-based surgical step recognition method and developed a user-assisted annotation tool. This auxiliary system significantly improves the accuracy and efficiency of annotation, demonstrating the potential of deep learning in optimizing the surgical video annotation process. These methods identify different stages of surgery by extracting spatial information from images. Still, their accuracy is low due to the neglect of temporal features during the surgical process, especially in complex surgical stages. However, from Tables 2, 3, and 4, it can be seen that despite the continuous evolution of research methods, almost all laparoscopic surgery phase recognition methods still retain the spatial information extraction module, namely the spatial model. Still, in most cases, other models are also combined. Next, we will analyze and summarize these studies’ most commonly used spatial models further.

##### 4.2.3.1 Spatial model analysis

According to the summary of deep learning models in Tables 2, 3 and 4, it is evident that most studies employ deep Convolutional Neural Network (CNN) architectures for feature extraction. Among them, the method proposed in reference (54) utilizes a gated capsule autoencoder model (Gcaps_TAE) for surgical phase recognition in laparoscopic videos. This method achieved an accuracy of 98.95% on the Cholec80 dataset, significantly outperforming other state-of-the-art methods. In this approach, the Inception model is adopted for spatial feature extraction. The Inception model, a type of CNN architecture, improves the model’s expressive power and computational efficiency by extracting multi-scale feature information by introducing convolutional kernels and pooling layers of various sizes. The application of CNN architecture in these studies can achieve excellent results due to the inherent characteristics of CNNs. CNNs use convolutional and pooling layers to extract features and reduce dimensionality from input images. It excels at capturing local image features and is particularly suitable for processing image data in laparoscopic surgery. Meanwhile, CNNs can effectively recognize and classify surgical instruments, tissue structures, etc., in images. These advantages enable CNN to perform excellently in both single-task and multi-task scenarios in laparoscopic surgery phase recognition.

The Residual Network (ResNet) is also a type of CNN that introduces residual connections based on traditional CNN, allowing the network to train deeper. Residual connections allow input information to bypass one or more layers and pass directly to subsequent layers. This can alleviate the gradient vanishing problem and enable deeper networks to train successfully, ultimately extracting higher-level image features. ResNet is widely used to investigate phase recognition in laparoscopic surgery. For example, in (5), the authors employed the SEResNet50 to extract high-level features of video frames, and the encoder of this method only relies on stage annotation for training without depending on other auxiliary information. Furthermore, Zhang et al. (56) used the Slow-Fast Temporal Modeling Network (SF-TMN) method for surgical phase recognition. They used ResNet in this method to extract spatial features from video frames. Numerous studies, including those that applied ResNet to extract spatial features, ultimately improved significantly. Generally, due to its deep network structure and residual connections, ResNet enhances the model’s performance by extracting high-level features from complex images.

Recently, researchers have adopted Transformer for spatial feature extraction. For example, Pan et al. (55) put forward the Swin Transformer to obtain multi-scale features. The Swin Transformer can process images of various scales by combining the benefits of CNN and Transformer. It uses an improved self-attention mechanism to extract spatial information from images. Furthermore, Swin Transformer applies the Shifted Window, which minimizes computational costs while processing high-resolution images and preserving the capacity to extract local and global features. This method not only retains the advantages of the Transformer model in capturing long-distance dependencies but also combines the strengths of CNN in local feature extraction, making the Swin Transformer performance in handling complex visual tasks. Its multi-scale feature extraction and self-attention mechanism enable the model to identify different phases in surgical videos accurately. For instance, the Swin Transformer enhances phase recognition accuracy by precisely identifying the usage of surgical instruments and changes in tissue structure when exploring high-resolution surgical videos.

##### 4.2.3.2 Temporal model analysis

The above summary outlines the commonly applied spatial models in laparoscopic surgery phase recognition. The widely used spatial models and their functions, along with the studies utilizing them, are organized in Table 5. However, depending solely on spatial features is insufficient for comprehensively understanding the dynamic changes during surgery. Therefore, with the advancement of technology, temporal models have gradually been introduced into surgical phase recognition, especially recurrent neural networks (RNNs) such as long short-term memory networks (LSTM) have been applied to the processing of surgical videos. These models can effectively capture the temporal dependencies between video frames, significantly improving recognition accuracy. For example, a hybrid model combining CNN for spatial feature extraction and LSTM for processing temporal information has gradually become the mainstream method. This type of method can better capture the dynamic characteristics of each stage during the surgical process and has achieved significant performance improvements in some studies. Standard temporal models include Long Short-Term Memory (LSTM), Temporal Convolutional Networks (TCN), and Transformer-based models. The following sections will offer a detailed analysis of these temporal models.

**Table 5:** Common spatial models used in studies related to surgical phase recognition tasks in laparoscopic surgery videos.

Architecture	Functions	Year	Methods
CNN (1998)	1. Image recognition and classification 2. Object detection 3. Image segmentation	2019	(24–28)
		2020	(1, 30–34)
		2021	(5, 10, 18, 24, 35, 38–40, 67)
		2022	(4, 7, 13, 19, 22, 43–46, 48)
		2023	(21, 54, 56, 57, 59, 61)
		2024	(63, 64)
ResNet (2015)	1. Image recognition and classification 2. Object detection 3. Image segmentation 4. Image generation	2019	(24, 29)
		2020	(1, 31, 33)
		2021	(35, 38)
		2022	(4, 19, 22, 43, 44, 46)
		2023	(21, 56, 61)
		2024	(63, 64)
Transformer (2017)	1. Image recognition and classification 2. Object detection 3. Image segmentation 4. Semantic segmentation	2021	(16)
		2022	(13)
		2023	(52, 53, 55, 56, 59, 62)
		2024	(64, 65)

The above-mentioned Gcaps_TAE (54) uses the Inception model and the 2D-LSTM models. The Inception model was adopted for extracting spatial features, while the 2D-LSTM model was utilized to extract temporal features, enabling the model to capture essential features better. Pan et al. (55) utilized a combination of Swin Transformer and LSTM. Swin Transformer is applied to extract multi-scale visual features, while LSTM is employed to extract temporal information from sequence frames. Through its gating mechanism, the LSTM can capture and remember long-term sequence information in surgical videos by merging the features that the Swin Transformer outputs. Therefore, LSTM is commonly used to extract temporal features in the phase recognition task of laparoscopic surgery. Typically, LSTM is used with CNNs or other spatial feature extraction models to obtain joint modeling of spatiotemporal features.

In addition to LSTM networks, TCNs and Transformers are commonly used temporal models in laparoscopic surgery phase recognition tasks. As mentioned earlier, Zhang et al. (56) proposed SF-TMN for surgical phase recognition. The proposed network operates in two stages: during the first stage, ResNet50 is used to extract spatial features from video frames; during the second stage, the extracted full video features are used for training, employing two different temporal modeling networks, Multi-Stage Temporal Convolutional Network (MS-TCN), and Transformer for Action Segmentation (ASFormer). The slow pathway of SF-TMN focuses on frame-level temporal modeling, while the fast pathway concentrates on segment-level temporal modeling. The initial predictions are generated by combining features from both the slow and fast pathways and are further optimized in a subsequent temporal refinement stage. Additionally, the proposed model achieves excellent results by combining TCN and Transformer for temporal feature extraction, with an evaluation accuracy of 95.43%.

TCNs excel in handling time series data. TCNs capture long-term dependencies through dilated convolution operations, making it adept at processing time-series data over extended periods. Dilated convolutions introduce gaps within the convolution operations, which can effectively expand the receptive field of the convolutional kernel without increasing computational complexity. This capability makes TCNs particularly effective for managing long-term dependencies in laparoscopic surgery videos, enabling it to capture critical dynamic changes during surgical procedures.

Based on attention mechanisms, Transformers can process entire time series data in parallel, providing efficient parallel computing capabilities suitable for handling very long sequences. With self-attention mechanisms, Transformers can consider information from all other time steps when computing the output for each time step. This makes them exceptionally good at capturing complex temporal dependencies. In laparoscopic surgery phase recognition tasks, Transformers are usually employed as temporal models to optimize methods. Transformers excel in capturing vital actions and stage transitions during surgical procedures.

##### 4.2.3.3 Model fusion and optimization strategy

The commonly used time models and their functions in laparoscopic surgery phase recognition tasks, as well as the studies with these models, are summarized in Table 6. The combination of spatial and temporal models has demonstrated strong performance in surgical phase recognition tasks. Combining different models can improve the accuracy of phase recognition. Apart from combining spatial and temporal models, some optimization strategies have also improved accuracy, such as attention mechanisms, residual connections, and data augmentation.

**Table 6:** Predominant temporal models in research on surgical phase recognition tasks for laparoscopic surgery videos.

Architecture	Functions	Year	Methods
LSTM (1997)	1. Time series prediction 2. Video analysis 3. Speech recognition 4. Natural language processing	2019	(24–26)
		2020	(34)
		2021	(10, 18, 23, 39)
		2022	(7, 19, 45, 48, 49)
		2023	(51, 55)
Transformer (2017)	1. Time series forecasting 2. Video analysis 3. Speech recognition 4. Natural language processing	2021	(16)
		2022	(13)
		2023	(52, 53, 55, 56, 59, 62)
		2024	(64, 65)
TCN (2018)	1. Time series forecasting 2. Speech recognition 3. Natural language processing 4. Action recognition	2020	(31)
		2021	(36, 38, 42)
		2022	(7, 22, 43, 44, 46, 49)
		2023	(50, 56, 57, 61, 62)
		2024	(63)

The attention mechanism can be used in laparoscopic surgery phase recognition to capture important temporal and spatial information by analyzing the relationship between different surgical video frames. By using the attention mechanism, the model can identify which frames are most important for the current surgical phase recognition, improving recognition accuracy. For example, in the method based on the Gcaps_TAE proposed in reference (54), to help the Inception model better learn important features in images, it is also integrated with the attention mechanism. By calculating the relationship between video frames, the attention mechanism focuses more on frames that are relatively important to the current task. This technique not only makes the model easier to identify but also makes it able to capture the tiny changes that occur during surgery.

Another widely used technique is residual connection, which allows input to skip one or more layers and pass directly to subsequent layers by adding shortcut connections between layers. In laparoscopic surgery phase recognition, deep neural networks must handle complex surgical videos, and residual connections can effectively train deeper networks to alleviate vanishing gradient problems and improve model performance. In reference (54), residual connections were added between the Inception model’s attention modules. Through residual connections, the output of the previous attention module is directly added to the output of the next module. During the training process, this method helps optimize the model by preserving the original features and improving the capacity of subsequent features to learn. Additionally, residual connections can enhance the ability to extract features by reducing the gradient vanishing.

Data augmentation is a technique for producing different training data through different random changes in the training data, including rotation and cropping. The primary purpose of this method is to improve the model’s generalization ability. Data augmentation can simulate different changes and uncertainties during the surgery. The model can be trained on various data types through data augmentation, which can better adapt to changes in practical applications. For example, in (20), researchers enriched the training dataset using data augmentation techniques, including color enhancement. These enhancement techniques enable the model to learn more features during the training process, improving the F1 score.

In recent years, with the diversification of medical data and the development of deep learning technology, researchers have begun to experiment with multimodal fusion methods. As mentioned above, Yuan et al. (22) improved the accuracy of surgical phase prediction through multimodal fusion methods. This method combines surgical videos with other types of data to further improve the accuracy of phase recognition, such as sensor data and audio data. Introducing force sensors, temperature sensors, and other data enables the model to integrate more dimensional information, thereby providing more accurate recognition results in complex surgical procedures.

In addition, deep transfer learning and federated learning have also been widely applied in laparoscopic surgery phase recognition. With the diversification of data and the demand for cross-device applications, deep transfer learning enables pre-trained models to adapt to data from different hospitals and devices, avoiding the difficulty of annotating surgical data. Transfer learning significantly improves the generalization ability of models by pre-training them on large-scale datasets and then fine-tuning them to adapt to specific data. For example, Daniel Neimark et al. explored in paper (23) how to improve the generalization performance of surgical step recognition through transfer learning across different surgical types. In addition, federated learning, as a privacy-preserving distributed training method, can train models across multiple hospitals or devices without centralized data storage, effectively protecting patient privacy and achieving good results in practical applications. For example, Hasan Kassem et al. proposed a Semi-Supervised Federated Learning (FSSL) method called Federated Cycling (FedCy) (21) for surgical stage recognition. FedCy is the first federated learning method applied to surgical videos, avoiding data-sharing issues.

##### 4.2.3.4 Summary

**Figure 4:**
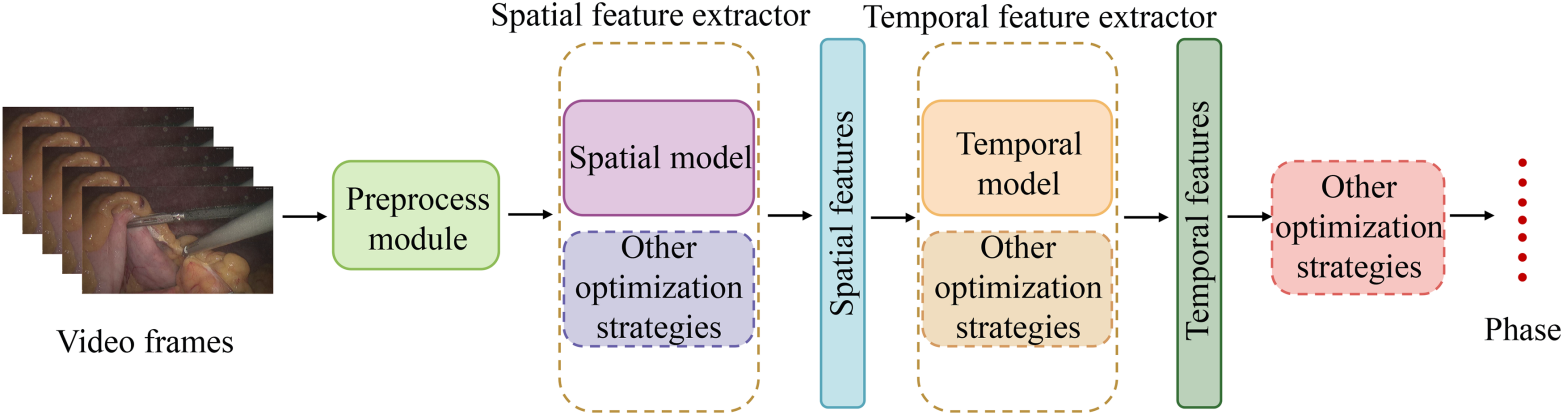
Commonly used method structures in laparoscopic surgery phase recognition research.

Based on the above analysis and the summary of Tables 2, 3 and 4, it can be concluded that most methods for the phase recognition task in laparoscopic surgery are based on the following architecture: the combination of spatial and temporal models and various optimization strategies. As shown in Figure 4, these optimization techniques are appropriate for both spatial and temporal models. Spatial models are mainly employed to extract spatial features from surgical videos, while temporal models capture dynamic changes during the surgery. Researchers can enhance phase recognition accuracy by merging spatial and temporal models. Similarly, optimization strategies, including attention mechanisms, residual connections, and data augmentation, can also enhance the model’s performance. These strategies improve the accuracy of feature extraction and address the problems that deep learning models may encounter during the training process, such as gradient vanishing and overfitting. We hope that through these analyses and summaries, we can help researchers in this field to overview the current research status and gain inspiration.

### 4.3 Applications of phase recognition

**Figure 5:**
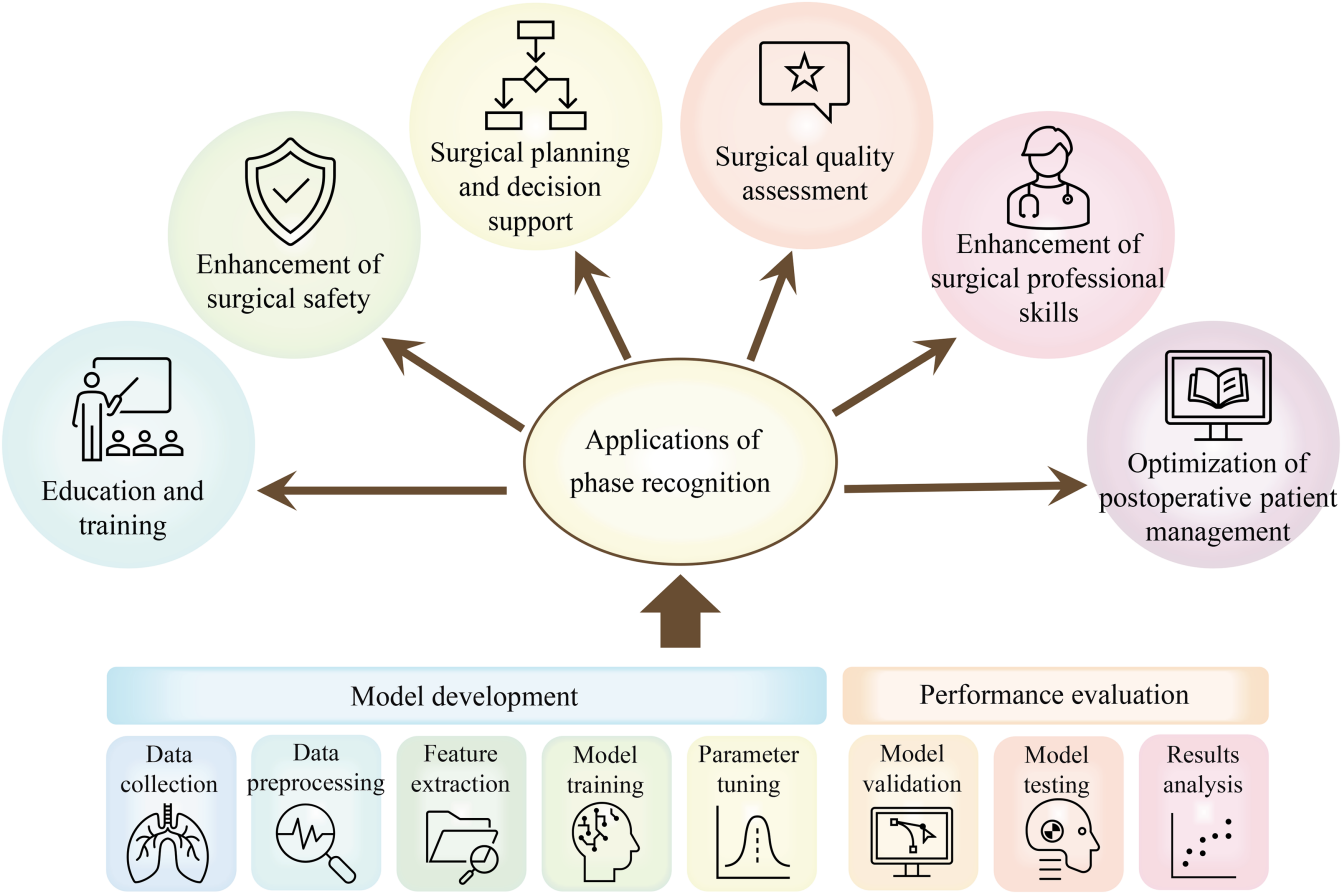
Applications of phase recognition tasks in laparoscopic surgery: including a concise process of surgical phase identification using DL models.

In the current section, we will analyze in detail the multiple application areas of the laparoscopic surgical phase recognition task, as presented in Figure 5. This task presents significant advantages during the surgical planning and evaluation phase. Phase recognition can help surgeons more accurately plan surgical steps and predict the time required for surgery. After the completion of the surgery, the surgeon’s key decisions and surgical operations are comprehensively evaluated (1), further improving the accuracy of surgical planning. During surgery, real-time phase recognition provides surgeons with immediate feedback to assist them in identifying the current stage of surgery and predicting the next operation, which not only enables surgeons to make more accurate and rapid decisions and avoid surgical errors (43) but also enables surgeons to prepare in advance through the system warning of upcoming complex surgical steps. This further improves the safety and success rate of surgery.

In addition, the surgical phase recognition task greatly supports surgical education (51). By automatically labeling the surgical stage, learners can more easily understand the entire surgical process, focus on the key skills in the surgical stage, and strengthen the learning and mastery of surgical operations. The analysis of the duration of different surgical stages and related operations can also be used to assess the surgeon’s surgical skills (51) and identify potential problems in the surgical process, which can improve the quality of the surgery and the surgeon’s professional skills. For novice surgeons, they can quickly learn surgical skills and discover their problems by observing and analyzing the surgical videos of skilled surgeons.

Retrospective identification of steps in surgical videos also exerts a vital role in postoperative patient care (50). Through these video analyses, surgeons can better understand the key aspects of postoperative care and ensure that patients receive the best postoperative care and treatment.

## 5 Research on laparoscopic surgery skill assessment

The treatment outcomes of patients are closely associated with surgeons’ surgical skills. Thus, it is essential to research surgical skill assessment, aiming to train surgeons and improve their surgical skills based on the feedback from the surgical skill assessment. The assessment of laparoscopic surgical skills is a complex process involving multiple standards and methods.

### 5.1 Standards and methods for surgical skill assessment

**Figure 6:**
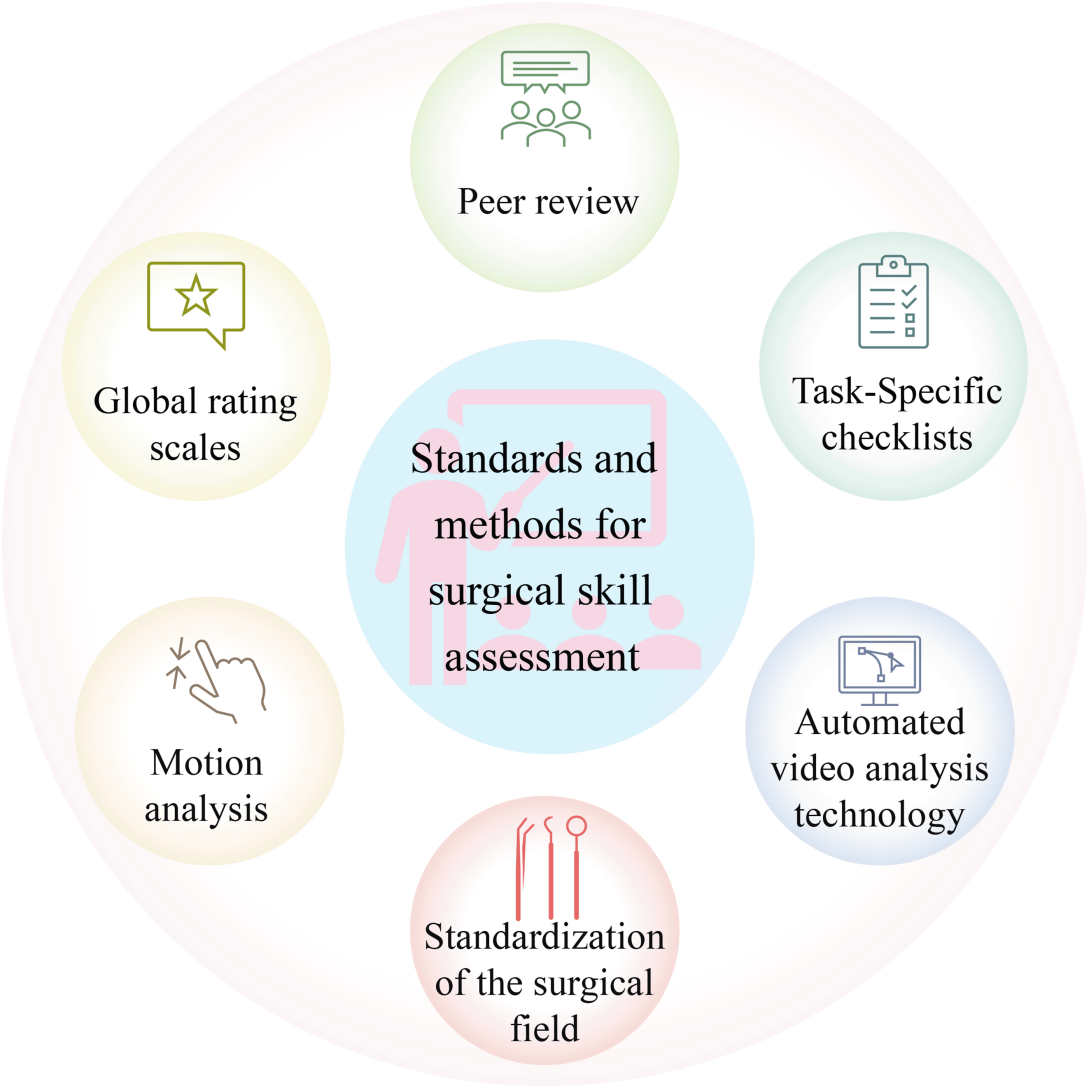
Common standards and methods for skill assessment in laparoscopic surgery.

The evaluation of laparoscopic surgical skills is mainly performed by analyzing surgical videos, which can provide a more intuitive observation of the surgeon’s operational skills during the surgical process. The key methods for evaluating surgical skills mainly consist of expert review, integration of motion recognition techniques, standardization of surgical field of view, and analysis of surgical instrument usage. In the laparoscopic surgery video skill assessment task, commonly used standards and methods can be observed in Figure 6, and these standards and methods will be detailed below.

Having surgical videos reviewed by experts is a relatively traditional evaluation method, where experts evaluate surgical skills based on their own experience and established standards. Although artificial intelligence is advancing, expert review is still vital for evaluating surgical skills. However, the expert review also has certain drawbacks, as it depends on personal experience and judgment, inevitably adding subjectivity (70).

Although expert review can effectively evaluate the skills of surgeons, its repeatability and accessibility are limited (71). In addition, this process is very time-consuming and laborious. Therefore, with the development of deep learning, researchers have shifted their attention to investigating automatic surgical skill assessment. Numerous studies analyze surgical tool movement through motion tracking to evaluate surgical skills (70). Studies have indicated that evaluating surgical skills through motion tracking can effectively distinguish the performance of expert surgeons from novice surgeons. Motion tracking mainly evaluates the flexibility of surgeons in operating surgical tools. This method provides objective data support, including the path length of surgical tools and the range of surgical tool movement (71), which can be adopted for analyzing the action economy and tool utilization efficiency during the surgical process. Moreover, combining action tracking and deep learning provides new possibilities for surgical skill assessment.

Studies have found that surgeons with different skill levels have different uses of surgical tools in laparoscopic surgery. Thus, the quantitative analysis of the use of tools in laparoscopic surgery is also a way of thinking in the task of laparoscopic surgical skill assessment (72). Studies have shown that during the knotting and suturing operations of laparoscopic surgery, the surgical movement data of surgeons with different surgical levels are different, such as the acceleration, angular velocity, and direction of the surgeon’s arm (73). Therefore, it is possible to evaluate the level of surgical skills of surgeons by analyzing sensor data during surgery (73). Clarity, stability, and control of coverage of the surgical field are also vital aspects in evaluating laparoscopic surgical skills. The development of appropriate surgical horizons can not only be applied to evaluate surgical skills but also exert a role in improving the safety of laparoscopic surgery (70).

### 5.2 Applications of surgical skill assessment

AI-based laparoscopic surgery video skill assessment methods provide a lot of advantages. At first, artificial intelligence can automatically analyze surgical videos using machine learning algorithms, providing more objective results than traditional manual evaluations. This lowers the subjective bias introduced by human evaluators and lightens their workload (70). Moreover, automated assessment can significantly shorten the evaluation time, enhance efficiency, and reduce costs.

**Figure 7:**
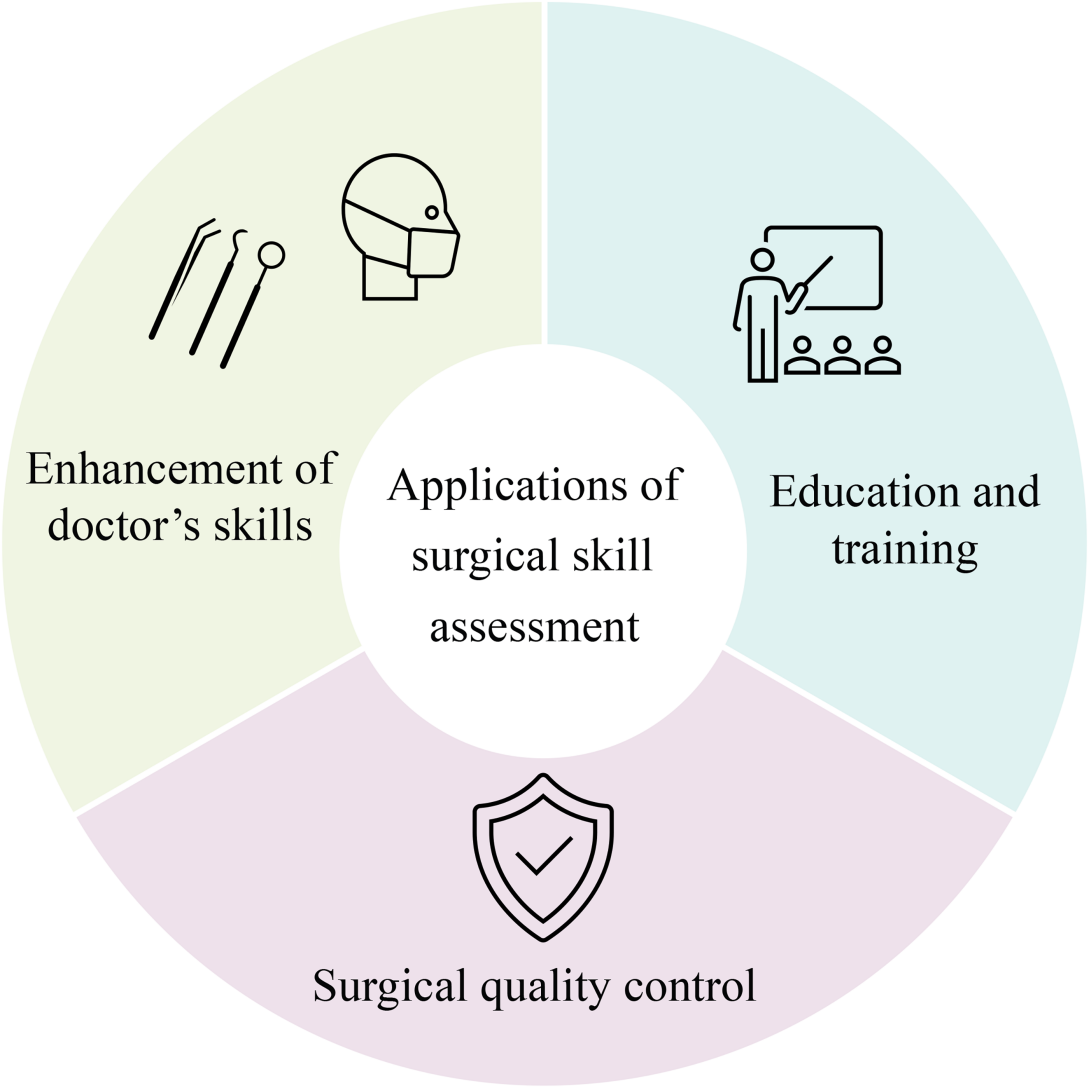
Overview of applications for skill assessment in laparoscopic surgery videos.

The application of surgical skill assessment mainly focuses on the following two perspectives: education and training of surgeons and ensuring surgical quality, as shown in Figure 7. By evaluating surgical skills, novice surgeons can learn based on videos of expert surgeons and corresponding evaluation results. Meanwhile, they can also discover and reflect on their shortcomings through their evaluation results and practice in a targeted manner. Regarding surgical quality assurance, regular evaluation of the surgical skills of surgeons in practice can ensure that surgeons have the essential skill level for surgery, timely identify surgeons with insufficient skills, and provide necessary training, significantly improving the success rate and safety of surgery (71).

## 6 Discussion

Research on stage recognition and skill evaluation in laparoscopic surgery presents several challenges, which can be categorized into two perspectives: *surgery-oriented challenges*, arising from the inherent complexities of the procedure, and *technology-oriented challenges*, related to the application of artificial intelligence technology.

Surgery-oriented challenges primarily stem from the complexities of the laparoscopic surgery environment. Factors such as bleeding, occlusion, smoke, and variations in operator habits often lead to poor visibility, overlapping of organs and instruments, and significant lighting changes in video footage. These issues can interfere with the input data for surgical stage recognition models, reducing their accuracy and robustness. Additionally, different types of laparoscopic procedures have unique characteristics in terms of organ anatomy, surgical techniques, and intraoperative challenges, making it difficult to develop a universal system for surgical stage recognition or skill evaluation.

Technology-oriented challenges arise from the application of artificial intelligence in surgical analysis. Data imbalance is a common issue, as surgical videos often emphasize certain frequent procedures, while some stages are brief and lack sufficient data, leading to model bias during training. Effectively leveraging temporal and spatial information is another key challenge, such as capturing contextual relationships between surgical stages in long-sequence videos and detecting interactions between surgical instruments and tissues in localized images. Additionally, model generalization remains a concern, as performance may be limited when applied to data from different hospitals, equipment, or surgeons. In few-shot learning scenarios, achieving robust stage recognition and skill evaluation with minimal labeled data is an urgent problem that needs to be addressed.

Beyond the challenges from the surgical and technological perspectives, it is also crucial to consider the new opportunities and challenges brought by emerging surgical equipment. For example, with the increasing adoption of robot-assisted surgical systems, stage recognition and skill evaluation must adapt to these advancements. On one hand, robotic systems offer a stable field of view, more precise instrument control, and automated recording capabilities, which may help reduce the complexity of stage recognition. On the other hand, multi-arm coordination, remote master-slave control, and the lack of direct tactile feedback introduce new challenges, such as increased instrument occlusion and dynamic changes in the operating environment. Additionally, variations in software versions, operational characteristics, and data formats across different robotic platforms can lead to domain shifts, making model generalization and cross-platform adaptation more difficult. Developing effective adaptation techniques between robotic and traditional laparoscopic systems remains a key direction for future research.

Despite these challenges, the rapid advancement of deep learning offers new approaches for processing complex surgical videos. Self-supervised learning enables models to leverage large amounts of unlabeled surgical videos, extracting richer features while reducing dependence on manual annotation. Additionally, ongoing research explores the integration of multimodal information, such as visual data and auxiliary signals, to enhance contextual understanding in surgical stage recognition and skill evaluation. Looking ahead, greater emphasis should be placed on real-time clinical deployment and multi-center, multi-scenario validation to ensure the stability and generalizability of AI systems across diverse practical environments.

Accurate surgical stage recognition and skill evaluation can enhance both intraoperative and postoperative outcomes. During surgery, it enables real-time process guidance and risk warnings, helping to reduce errors. After surgery, it provides objective skill assessment and personalized training programs for surgeons. For patients, precise stage identification and standardized surgical procedures contribute to shorter recovery times and a lower risk of complications. With continued advancements in multimodal data fusion, deep learning, and clinical validation, the research on automated stage recognition and skill evaluation in laparoscopic surgery holds great promise for broader clinical applications.

## 7 Conclusion

This study reviews recent research in laparoscopic phase recognition and skill assessment. As an advanced minimally invasive surgical technique, laparoscopic surgery requires very high surgical skills. Therefore, phase recognition is crucial to evaluating surgical skills and improving surgeons’ skills.

Through detailed analysis of publicly available datasets, including Cholec80, M2CAI16-workflow, and AutoLaparo, we lay the foundation for the study of laparoscopic surgery phase recognition research conducted on public datasets. We summarize the structures of models that have exhibited strong performance in this task, detailing commonly used spatial models, temporal models, and other optimization strategies. Many of these methods have achieved promising results.

However, this field of research still confronts several challenges, including handling complex scene variations in surgical videos, addressing occlusions of surgical tools, and learning automatically from large-scale unannotated video data. In addition, current research mainly focuses on specific types of laparoscopic surgeries, lacking extensive studies on different surgical types.

From a practical application perspective, implementing phase recognition and skill assessment technologies in clinical practice requires overcoming challenges related to data privacy, algorithmic interpretability, and integration with existing medical systems. Moreover, introducing these technologies must consider their acceptance by medical professionals, ensuring that surgeons widely recognize the technology’s practicality and effectiveness.

In conclusion, despite the existing challenges, the research and application of phase recognition and skill assessment technologies in laparoscopic surgery demonstrate substantial development potential. With constant technological advancements and deeper integration with medical practice, significant progress is expected to be made in ensuring surgical quality, enhancing surgical training, and assessing surgical skills in the future.
